# Case Report: Multiple intra-abdominal lymph node abscesses due to *Staphylococcus aureus* in a cat

**DOI:** 10.3389/fvets.2025.1654990

**Published:** 2025-11-17

**Authors:** Kei Tamura, Kazuya Kushida, Tomoko Iwanaga, Ryuji Fukushima, Keitaro Ohmori

**Affiliations:** 1 Koganei Animal Medical Emergency Center, Tokyo University of Agriculture and Technology, Tokyo, Japan; 2 Research Unit of Innovative One Medicine, Advanced Research Center for One Welfare, Tokyo University of Agriculture and Technology, Tokyo, Japan

**Keywords:** abscess, antibiotic, cat, intra-abdominal lymph node, *Staphylococcus aureus*

## Abstract

Intra-abdominal lymph node abscesses (LAs) are rare in cats and have previously been reported only in the mesenteric lymph nodes, caused by *Escherichia coli* or *Listeria monocytogenes*. The full spectrum of causative agents and the underlying pathogenesis remain poorly understood. A 4-year-old spayed indoor cat presented with a one-week history of fever and anorexia. Laboratory tests revealed marked neutrophilia, eosinophilia, and an elevated feline serum amyloid A level. Computed tomography identified cyst-like masses in the medial iliac and hepatic lymph nodes. Cytological analysis showed infiltration by neutrophils and macrophages without neoplastic cells. Gram-positive cocci were observed, and *Staphylococcus aureus* was isolated from the abscesses. Based on antimicrobial susceptibility testing, amoxicillin was selected as the treatment. Administration of amoxicillin led to clinical improvement and normalization of hematological abnormalities. Antibiotic therapy was continued for approximately 6 months, after which no recurrence was observed. The cat remained in good health at 689 days after the initial presentation. This is the first reported case of multiple intra-abdominal LAs caused by *S. aureus* in a cat. The case highlights the pathogenic potential of this commensal bacterium and underscores the importance of long-term, susceptibility-guided antibiotic therapy in achieving complete resolution.

## 1 Introduction

Intra-abdominal abscesses have been reported in the liver, kidney, and pancreas in cats (1–3). Fever and leukocytosis are typical clinical features of this condition (1–3). Previous case reports have described intra-abdominal lymph node abscesses (LAs) involving the mesenteric lymph nodes in cats (4, 5). *Escherichia coli* and *Listeria monocytogenes* were identified as the causative pathogens in these cats (4, 5). However, other potential pathogens responsible for intra-abdominal LAs in cats remain unclear.

Intra-abdominal LAs have also been reported in dogs, affecting the mesenteric, retroperitoneal, and iliac lymph nodes (6–9). In contrast to cats, multiple bacterial species, such as *E. coli*, *Staphylococcus aureus, S. epidermidis*, *S. canis, Serratia marcescens, Prevotella* species, and *Salmonella* species, have been implicated in dogs (6, 7). Chronic gastrointestinal inflammation, sepsis, or peritonitis may be associated with the development of intra-abdominal LAs in dogs (9, 10). However, whether the underlying pathogenesis is similar between dogs and cats remains unclear.

Herein, we report for the first time that *S. aureus* induced multiple intra-abdominal LAs in a cat. The cat was successfully treated with amoxicillin and achieved long-term survival.

## 2 Case description

**Figure 1:**
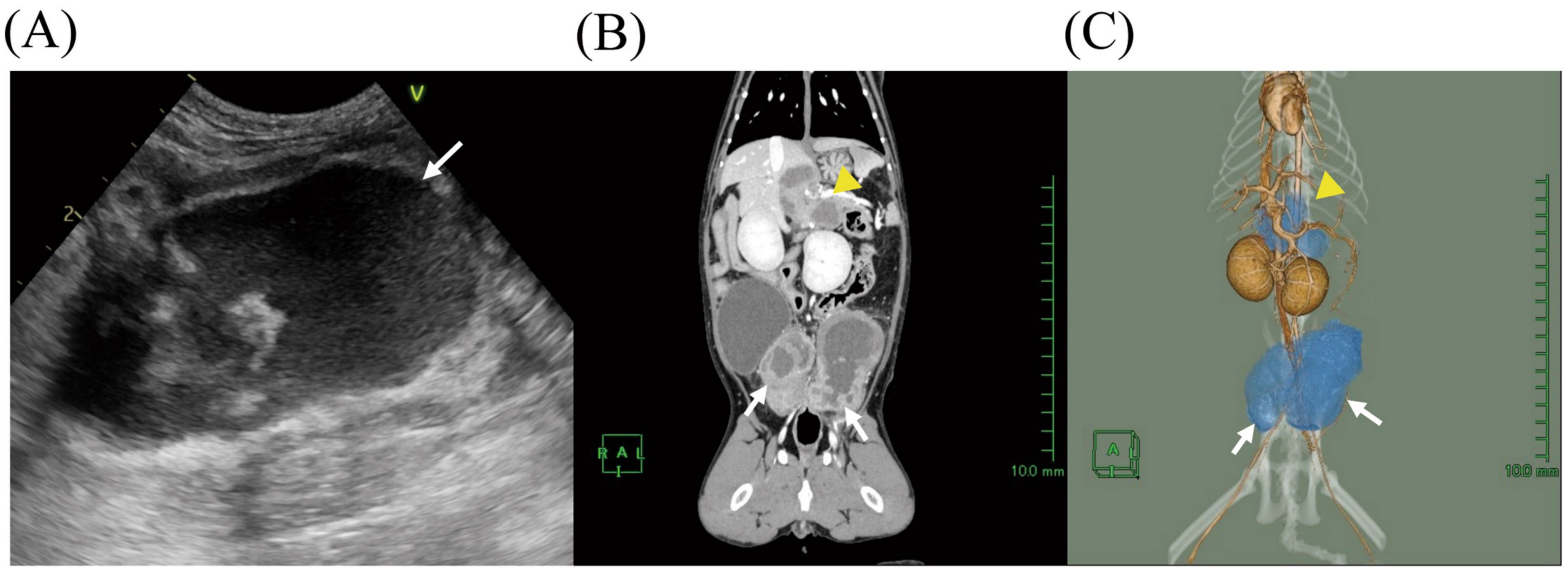
Ultrasound and contrast-enhanced computed tomography (CT) images of multiple intra-abdominal lymph node abscesses in a cat. **(A)** Abdominal ultrasound image of the medial iliac lymph nodes on day 1. **(B)** Contrast-enhanced coronal CT image and **(C)** three-dimensional (3D) CT reconstruction on day 2. Yellow arrowheads indicate the hepatic lymph nodes, and white arrows point to the medial iliac lymph nodes. In the 3D image, lymph nodes are shown in blue.

A 4-year-old spayed indoor mixed-breed cat weighing 2.78 kg (body condition score: 2/5) was presented to Koganei Animal Medical Emergency Center at Tokyo University of Agriculture and Technology with a one-week history of anorexia and lethargy (day 1). No gastrointestinal signs, such as vomiting or diarrhea, were observed. Physical examination revealed fever (40.0 °C) and weight loss. The cat had no prior medical history and had not received any medications, including glucocorticoids or immunosuppressive agents. A complete blood count (CBC) revealed leukocytosis [29.4 × 10^9^ cells/L; reference interval (RI), 5.5–19.5 × 10^9^ cells/L], characterized by increases in band neutrophils (4.3 × 10^9^ cells/L; RI, 0–0.7 × 10^9^ cells/L), segmented neutrophils (19.1 × 10^9^ cells/L; RI, 2.5–12.5 × 10^9^ cells/L), and eosinophils (5.8 × 10^9^ cells/L; RI, 0–1.5 × 10^9^ cells/L). Serum biochemical analysis showed a marked elevation in feline serum amyloid A (f-SAA) level (71.8 μg/mL; RI, 0–6.8 μg/mL). A blood test for FIV and FeLV was negative. Thoracic radiography revealed no significant abnormalities. Abdominal ultrasonography identified heterogeneous, hypoechoic masses located near the liver and urinary bladder (Figure 1A). Urinalysis showed no abnormalities, including evidence of bacterial or fungal infection.

**Figure 2:**
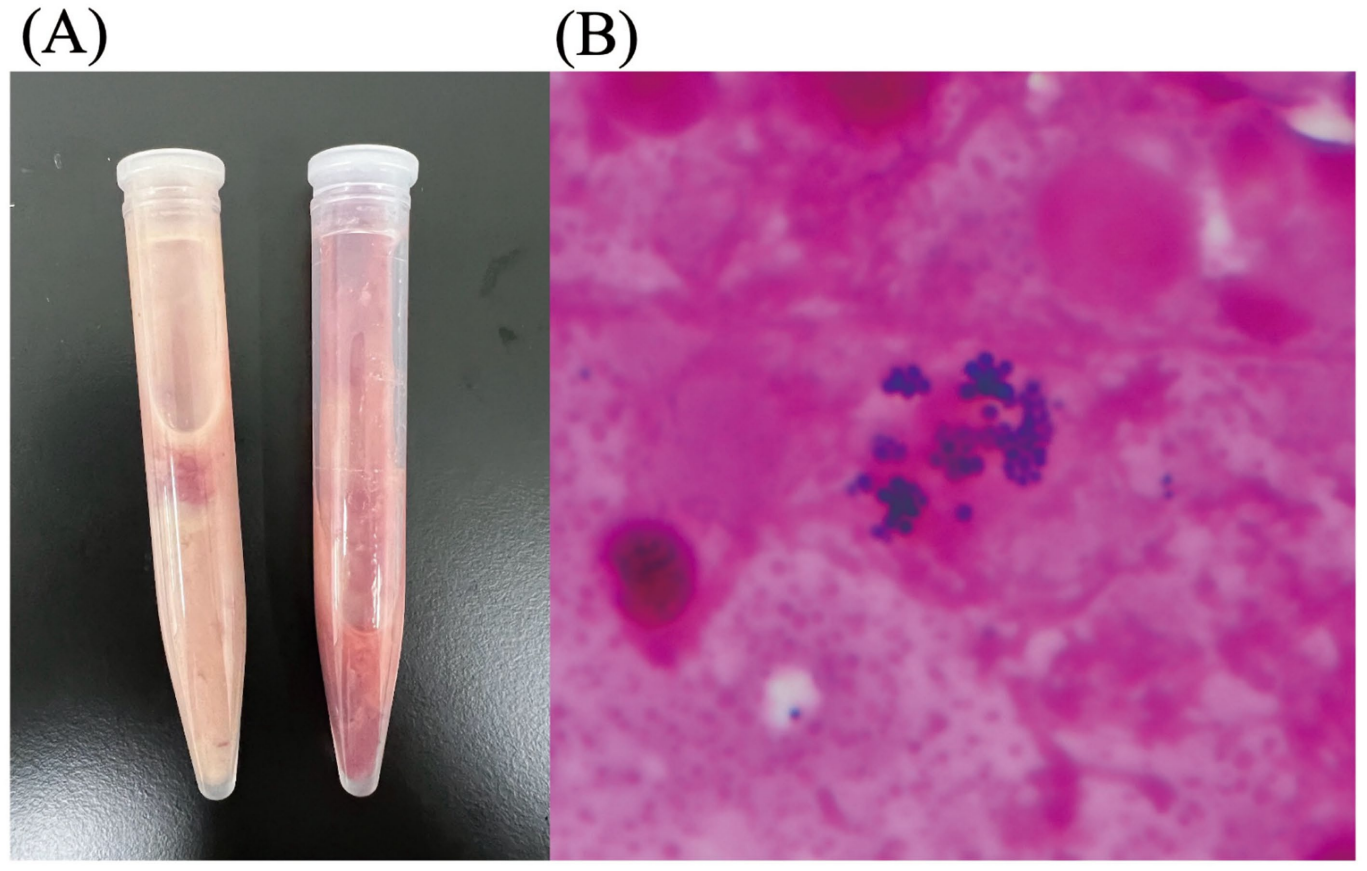
Aspirated fluid and Gram staining findings from intra-abdominal lymph node abscesses in a cat. **(A)** Milky white fluid collected from the intra-abdominal lymph node abscesses. **(B)** Gram-stained smear of the aspirated fluid showing gram-positive cocci.

To further evaluate the intra-abdominal masses, computed tomography (CT) was performed under general anesthesia on day 2. CT imaging revealed enlargement of the left medial iliac lymph node (35 × 36 × 58 mm; length × width × height), right medial iliac lymph node (19 × 23 × 45 mm), right hepatic lymph node (22 × 13 × 31 mm), and left hepatic lymph node (15 × 12 × 18 mm). These lymph nodes exhibited cyst-like structures with perinodal contrast enhancement and heterogeneous fluid accumulation [30–60 Hounsfield units (HU)] in their central regions (Figures 1B,C), resulting in stenosis of the descending colon and compression of the left deep circumflex iliac artery and vein. Fine-needle aspiration (FNA) of the enlarged medial iliac and hepatic lymph nodes was performed under ultrasound guidance after shaving and disinfecting the puncture site, yielding milky white fluid (Figure 2A). Cytological analysis of the aspirate demonstrated infiltration by neutrophils and macrophages but did not reveal any neoplastic cells, including lymphoma. Gram staining of the fluid identified gram-positive cocci (Figure 2B). Based on these findings, the enlarged medial iliac and hepatic lymph nodes were diagnosed as abscesses. The FNA samples from the medial iliac and hepatic LAs were submitted for bacterial culture, species identification, and antimicrobial susceptibility testing. These analyses were performed through the Antibacterial Tests for Animals service (1 sec. Co., Ltd., Kanagawa, Japan). Species identification was performed using Matrix-Assisted Laser Desorption/Ionization Time-Of-Flight Mass Spectrometry (MALDI-TOF MS) with the MALDI Biotyper® sirius one System (Bruker Corporation, Billerica, MA, USA). In addition, colonoscopy was performed to investigate the underlying cause of the medial iliac LAs. However, histopathological examination of the colonic mucosa revealed no abnormalities.

The cat was hospitalized and treated from day 2 to day 8. Based on the Gram staining results of the intra-abdominal LAs, empirical antibiotic therapy was initiated before the results of bacterial culture and antimicrobial susceptibility testing were available. The cat received intravenous cefazolin (30 mg/kg; Kyoritsu Seiyaku Co., Ltd., Tokyo, Japan) twice daily. However, this treatment did not improve the fever or reduce the size of the intra-abdominal LAs on ultrasound examination. On day 6, *S. aureus* was identified by bacterial culture and species identification testing of the medial iliac and hepatic LAs. Given the results of the antimicrobial susceptibility test (Table 1) and the poor clinical response to cefazolin, the antibiotic was switched to amoxicillin. Intravenous amoxicillin (20 mg/kg; Ampiclear, Riken Vets Pharma, Saitama, Japan) was administered twice daily starting on day 6. By day 8, treatment with amoxicillin had reduced the cat’s body temperature (38.4 °C) and improved its lethargy and anorexia, leading to discharge. On the same day, oral administration of amoxicillin (20 mg/kg; Amoxiclear, Riken Vets Pharma) was initiated twice daily.

**Table 1:** Antimicrobial susceptibility test results for *Staphylococcus aureus* isolated from intra-abdominal lymph node abscesses in a cat.

Antibiotics	Result	Inhibition zone diameter (mm)	Criteria		
			S (mm)	I (mm)	R (mm)
Ampicillin	S	38	≥18	–	≤17
Amoxicillin/clavulanic acid	S	40	≥20	–	≤19
Cefalexin	S	24	≥18	15–17	≤14
Cefotaxime	S	22	≥21	18–20	≤17
Ceftriaxone	S	23	≥21	14–20	≤13
Imipenem	S	44	≥16	14–15	≤13
Faropenem	S	30	≥16	13–15	≤12
Enrofloxacin	S	29	≥23	17–22	≤16
Orbifloxacin	S	27	≥23	18–22	≤17
Amikacin	S	21	≥18	–	≤17
Erythromycin	S	26	≥23	14–22	≤13
Doxycycline	S	23	≥16	13–15	≤12

S, susceptible; I, intermediate; R, resistant (Criteria was based on Clinical and Laboratory Standards Institute, 2009).

On day 20, the cat was in good condition following the initiation of amoxicillin treatment. Abdominal ultrasonography showed reductions in the size of the hepatic and medial iliac LAs. A CBC revealed a decrease in white blood cells (10.8 × 10^9^ cells/L), including band neutrophils (0 × 10^9^ cells/L), segmented neutrophils (3.5 × 10^9^ cells/L), and eosinophils (2.1 × 10^9^ cells/L). Furthermore, the f-SAA level had returned to within the RI (<3.0 μg/mL).

**Figure 3:**
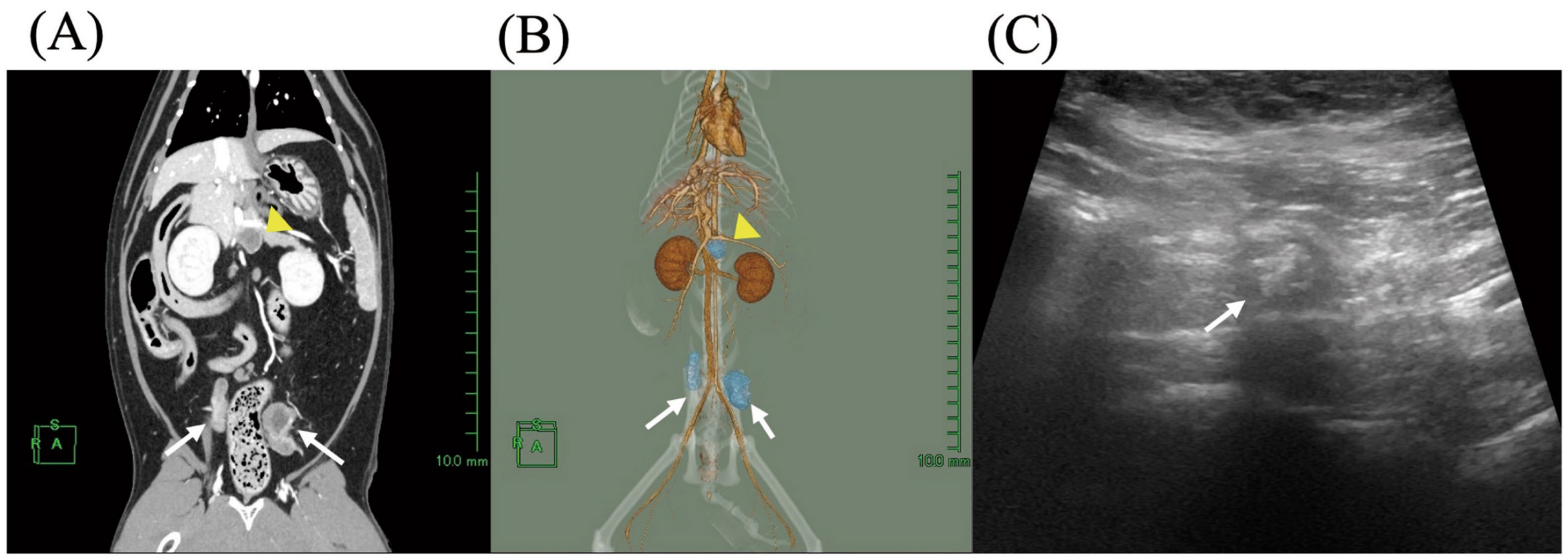
Follow-up imaging of intra-abdominal lymph node abscesses during antibiotic therapy in a cat. **(A)** Contrast-enhanced coronal computed tomography (CT) image and **(B)** three-dimensional (3D) CT reconstruction on day 90. **(C)** Abdominal ultrasound image of the medial iliac lymph nodes on day 132. Yellow arrowheads indicate the hepatic lymph nodes, and white arrows indicate the medial iliac lymph nodes. In the 3D image, lymph nodes are shown in blue.

On day 90, a follow-up CT scan performed under general anesthesia revealed reductions in the size of the intra-abdominal LAs, including the left medial iliac lymph node (10 × 12 × 14 mm), right medial iliac lymph node (8 × 7 × 6 mm), right hepatic lymph node (7 × 6 × 10 mm), and left hepatic lymph node (12 × 12 × 10 mm) (Figures 3A,B). However, as fluid retention within the LAs was still evident, amoxicillin treatment was continued. On day 132, abdominal ultrasonography showed further reductions in the size of the LAs and no remaining fluid accumulation (Figure 3C). Amoxicillin was maintained until day 182 to prevent recurrence. After day 182, the cat received no further medication. At the final follow-up on day 689, the cat remained in good condition without clinical signs or recurrence. Furthermore, ultrasonography revealed no lymph node enlargement, indicating complete resolution of the intra-abdominal LAs.

## 3 Discussion

This is the first case report to describe *S. aureus*-induced multiple intra-abdominal LAs involving the medial iliac and hepatic lymph nodes in a cat. In addition, treatment with amoxicillin resulted in complete resolution of the intra-abdominal LAs, clinical signs, and clinicopathological abnormalities. The findings of this case highlight the pathogenic potential of *S. aureus* in feline intra-abdominal LAs and underscore the importance of appropriate antibiotic therapy in managing this condition in cats.

*Staphylococcus aureus* has been identified as a cause of intra-abdominal LAs in dogs (6). Although intra-abdominal *S. aureus* infection has previously been reported in the pancreas of a cat (3), involvement of the hepatic and medial iliac lymph nodes has not been described in cats. Intra-abdominal abscesses in cats have been associated with cholecystitis, digestive tract disorders, or cystitis (1–3, 11). In this case, CT imaging did not reveal any lesions indicative of *S. aureus* infection in organs other than the intra-abdominal lymph nodes. Therefore, the route of *S. aureus* entry into the abdominal cavity and the underlying pathogenesis of intra-abdominal lymph node abscesses remain unclear in this cat.

Eosinophilia, together with neutrophilia and an elevated f-SAA level, was observed in this case. Notably, treatment with amoxicillin led to improvement of both the intra-abdominal LAs and eosinophilia, suggesting a potential association between them. Eosinophilia is a clinicopathological finding commonly associated with parasitic infections, allergic diseases, and certain tumors, such as mast cell tumors and bladder transitional cell carcinoma in cats (12–14). Recent studies have also implicated bacterial infections, including those caused by *Staphylococcus* species, in the development of feline gastrointestinal eosinophilic sclerosing fibroplasia (FGESF) (15, 16). Peripheral eosinophilia has been reported in approximately 50% of cats diagnosed with FGESF. These findings support the hypothesis that eosinophilia may occur in response to intra-abdominal bacterial infections, although the underlying mechanisms remain unclear. Eosinophilia may represent a relevant hematological abnormality associated with intra-abdominal LAs in cats. Further studies involving larger cohorts are needed to clarify the clinical significance of this observation.

Treatment of intra-abdominal LAs in dogs includes antibiotic administration, surgical resection, and drainage (6). In this case, surgical resection was not performed because the abscesses involved multiple lymph nodes and compressed the left deep circumflex iliac artery and vein. Therefore, amoxicillin was selected as an appropriate antibiotic based on the results of antimicrobial susceptibility testing and was administered to the cat, resulting in a successful clinical outcome. No recurrence of intra-abdominal LAs was observed following the discontinuation of antibiotic therapy. However, in this case, approximately 6 months of amoxicillin administration was required for complete resolution. These findings suggest that long-term antibiotic therapy may be necessary for cats with surgically non-resectable intra-abdominal LAs. Given that a previous report documented recurrence of intra-abdominal LA in a cat even after an extended course of antibiotic therapy (4), careful long-term follow-up is essential in managing this condition.

There are several limitations in this case report. *S. aureus* was identified using MALDI-TOF MS, which has been shown to accurately identify bacteria at the species level (17). However, molecular identification methods, such as PCR or 16S rRNA gene sequencing, were not performed. Therefore, the possibility that other bacteria, such as *Nocardia*, were involved in the development of intra-abdominal LAs cannot be completely ruled out. In addition, even though the LA sample for bacterial identification was collected after shaving and disinfecting the puncture site, it is plausible that *S. aureus* might have been a skin contaminant introduced during the procedure. However, *S. felis* and *S. epidermidis* are more commonly found *Staphylococcus* species in feline skin flora (18). Thus, the isolation of *S. aureus* from this sample may not be fully explained by normal skin contamination alone. Considering the nature of a case report, the clinical significance of *S. aureus* in intra-abdominal LAs in cats warrants further investigation in a larger cohort.

In conclusion, this case report provides evidence that *S. aureus*, a commensal bacterium, can act as a pathogen causing multiple intra-abdominal LAs in a cat. Long-term administration of amoxicillin, selected based on antimicrobial susceptibility testing, was crucial for the treatment of the cat. Further studies are warranted to elucidate the underlying pathogenesis of intra-abdominal LAs in cats.

## Data Availability

The data analyzed in this study is subject to the following licenses/restrictions: due to confidentiality and ethical considerations regarding the animal and owner, the dataset cannot be publicly shared. Requests to access these datasets should be directed to KO, k-ohmori@cc.tuat.ac.jp.
